# A new approach for sample size calculation in cost-effectiveness studies based on value of information

**DOI:** 10.1186/s12874-018-0571-1

**Published:** 2018-10-22

**Authors:** Clément Bader, Sébastien Cossin, Aline Maillard, Antoine Bénard

**Affiliations:** 1 CHU Bordeaux, Pôle de santé publique, Service d’information médicale, USMR & CIC 1401 EC (Clinical Epidemiology), F-33000 Bordeaux, France; 2 Inserm, Bordeaux Population Health Research Center, team EMOS, UMR 1219, University Bordeaux, F-33000 Bordeaux, France

**Keywords:** Cost-benefit analysis, Sample size, Value of information, Clinical trials, Comparative studies, Epidemiologic methods

## Abstract

**Background:**

Value of information is now recognized as a reference method in the decision process underpinning cost-effectiveness evaluation. The expected value of perfect information (EVPI) is the expected value from completely reducing the uncertainty surrounding the cost-effectiveness of an innovative intervention.

Among sample size calculation methods used in cost-effectiveness studies, only one is coherent with this decision framework. It uses a Bayesian approach and requires data of a pre-existing cost-effectiveness study to derive a valid prior EVPI. When evaluating the cost-effectiveness of innovations, no observed prior EVPI is usually available to calculate the sample size.

We here propose a sample size calculation method for cost-effectiveness studies, that follows the value of information theory, and, being frequentist, can be based on assumptions if no observed prior EVPI is available.

**Methods:**

The general principle of our method is to define the sampling distribution of the incremental net monetary benefit (Δ*B*), or the distribution of Δ*B* that would be observed in a planned cost-effectiveness study of size *n*. Based on this sampling distribution, the *EVPI* that would remain at the end of the trial (*EVPI*_*n*_) is estimated. The optimal sample size of the planned cost-effectiveness study is the *n* for which the cost of including an additional participant becomes equal or higher than the value of the information gathered through this inclusion.

**Results:**

Our method is illustrated through four examples. The first one is used to present the method in depth and describe how the sample size may vary according to the parameters’ value. The three other examples are used to illustrate in different situations how the sample size may vary according to the ceiling cost-effectiveness ratio, and how it compares with a test statistic-based method. We developed an R package (EBASS) to run these calculations.

**Conclusions:**

Our sample size calculation method follows the value of information theory that is now recommended for analyzing and interpreting cost-effectiveness data, and sets the size of a study that balances its cost and the value of its information.

**Electronic supplementary material:**

The online version of this article (10.1186/s12874-018-0571-1) contains supplementary material, which is available to authorized users.

## Introduction

When analyzing cost-effectiveness data, it is argued that rules of inference are arbitrary and entirely irrelevant to the decisions which clinical and economic evaluations claim to inform. When a choice is to be made between two interventions, a statistical test is of no use if its result is not significant. Indeed, a statistically insignificant difference at the level of a study sample cannot exclude a major difference at the level of a population. Furthermore, when the difference between interventions is statistically significant, the *p-value* quantifies the risk that there is truly no difference between interventions at the level of the population, a situation where choosing one of the compared interventions will have no consequence [1]. The decision process underpinning cost-effectiveness evaluation should be based only on the mean net benefits of each intervention irrespective of whether the difference between them is statistically significant [2].

The net monetary benefit (*B*) of an intervention is given by *E* × *λ* − *C*, where *E* and *C* are the effect and cost of this intervention, and λ is the threshold monetary value for a unit of effect.

When evaluating the cost-effectiveness of a new intervention in comparison with the reference, one can estimate the difference between the net monetary benefit of the new intervention (*B*_*N*_) and the net monetary benefit of the reference (*B*_*R*_). This difference is the incremental net monetary benefit (*∆B*) [3].

The uncertainty surrounding the decision based on the incremental net monetary benefit can be expressed through the risk of making the wrong decision or the risk of *∆B* being negative when its point estimate is positive, or vice versa. The expected value from completely reducing this uncertainty can then be defined as the expected opportunity loss when making the wrong decision, a situation which could be avoided having perfect information [4]. This is known as the expected value of perfect information (EVPI). The EVPI is used as a necessary requirement for determining the profitability of further research: additional research should be considered only if the EVPI exceeds the expected cost of further research [2, 5].

Comparative studies, including clinical trials, are commonly used as a vehicle for health economic evaluations [6]. A key aspect in the elaboration of such studies is the sample size calculation. It must yield sufficient precision in the estimations, and has ethical implications as it is unnecessary to expose too many individuals to the constraints of a clinical study [7]. As expected, the sample size calculation should be in agreement with the statistical methods planned to use for the data analysis [8].

Various sample size calculation methods have been developed for trial-based cost-effectiveness analyses. Most of them are based on test statistics comparing *∆B* to zero or the incremental cost-effectiveness ratio to *λ*, with assumption of asymptotic normality [9, 10] or by simulation [11]. Another sample size calculation method is based on a Bayesian framework for value of information analysis [12]. In this Bayesian framework, the first step is to estimate the difference between the posterior EVPI and the prior EVPI (i.e.: the expected value of sample information, EVSI). The EVSI is calculated at the level of a population of size *N* and depends on the number of individuals (*n*) to be included in the planned cost-effectiveness study. The difference between the EVSI and the cost of the planned study is the expected net gain (ENG) of further research. If, for a given *n*, the ENG is positive (i.e.: EVSI>cost of the trial) the planned study is considered useful and the optimal sample size of the planned study is the *n* that maximizes ENG.

Methods based on test statistics are not coherent with the decision process underpinning cost-effectiveness evaluation which does not support the use of a statistical test to compare the net monetary benefit between each intervention [2]. The main limitation of the Bayesian value of information framework is that determining the prior EVPI requires data of a pre-existing cost-effectiveness study in the same target population to derive a valid a priori distribution of the incremental net monetary benefit. Indeed, even if considering an uninformative prior is theoretically feasible, no such extension of their method has been proposed by the authors. Unfortunately, when evaluating the cost-effectiveness of innovations, no observed *prior* distribution of the incremental net monetary benefit is usually available to calculate the sample size.

We here propose a new sample size calculation method that follows the same principles as the Bayesian approach proposed by Willan et al., but using a frequentist approach. Our frequentist approach determines the optimal sample size by comparing the decrease in the expected EVPI due to the inclusion of additional participants to the cost of including more participants. It can be based on assumptions if a prior distribution of the incremental net monetary benefit from a pre-existing study involving the same population is not available.

## Methods

We primarily developed our method for situations where current information is lacking to guide decision making. A context where a cost-effectiveness study has to be conducted and where no decision can be drawn before the results are available. This context has two major implications for our method: 1) fixed costs, those required for setting up the study before patients’ recruitment, are considered mandatory and do not enter in our calculations; 2) If no prior information is available, the expected value of information will not be estimated before the results of the planned cost-effectiveness study are available. *N*, the size of the population to be considered in our calculation, is the number of individuals when a decision will be made, that is at the end of the study. Thus, the number of individuals recruited in the study is not taken into account in the calculation of the population EVPI.

In a first approach, we develop the case of a planned randomised trial-based cost-effectiveness analysis (CEA) with two parallel groups of equal size *n*/2 and equal variance of cost and effect.

The case of unequal variance of costs (${\sigma}_C^2$) and effect (${\sigma}_E^2$) in each group, and the case of unequal group size are further detailed in the Additional file 1.

*The three steps of our calculation method are as follows.*

A. Defining the sampling distribution of ∆B.

Let $$ \widehat{\boldsymbol{\Delta }B} $$ be the estimate of ***∆****B* in a planned randomised trial-based CEA of size *n*. Being a difference of differences, $$ \widehat{\boldsymbol{\Delta }B} $$ is a linear expression that may take all possible values from −∞ to +∞. As reported elsewhere [3, 12–15], we assume that $$ \widehat{\boldsymbol{\Delta }B} $$ follows a Normal distribution of mean *μ*_***∆****B*_ and variance $$ \raisebox{1ex}{$2{\sigma}_{\Delta  B}^2$}\!\left/ \!\raisebox{-1ex}{$n$}\right. $$.

When comparing two independent groups (i.e.: a randomised clinical trial): ${\sigma}_{\Delta  B}^2=2{\sigma}_B^2$, where ${\sigma}_B^2$ is the common variance of the net monetary benefit (*B*) in each group. The variance of *B* is given by:

1 $$ {\sigma}_B^2={\lambda}^2{\sigma}_E^2+{\sigma}_C^2-2{\lambda \rho \sigma}_E{\sigma}_C $$

Where ${\sigma}_C^2$ and ${\sigma}_E^2$ are the expected common variances of cost and effect in potential samples, and *ρ* the correlation between cost and effect [3].

Hence $$ \widehat{\mathbf{\Delta }B}\sim \mathcal{N}\left({\mu}_{\mathbf{\Delta }B},\raisebox{1ex}{$4\left({\lambda}^2{\sigma}_E^2+{\sigma}_C^2-2\lambda \rho {\sigma}_E{\sigma}_C\right)$}\!\left/ \!\raisebox{-1ex}{$n$}\right.\right) $$

B. Estimating the remaining value of perfect information for a defined sample size (*EVPI*_*n*_).

Taking into account $\widehat{\Delta  B}$, the value of perfect information that would remain after completing the planned randomised trial-based CEA of *n* participants is:

2 $$ {EVPI}_n=N\left[I\left({\mu}_{\Delta B}>0\right){\int}_{-\infty}^0-b\widehat{f}(b) db+I\left({\mu}_{\Delta B}\le 0\right){\int}_0^{+\infty }b\widehat{f}(b) db\right] $$

Where $$ \widehat{f} $$ is the density function for $$ \widehat{\Delta  B}\sim \mathcal{N}\left({\mu}_{\boldsymbol{\Delta }B},\raisebox{1ex}{$4{\sigma}_B^2$}\!\left/ \!\raisebox{-1ex}{$n$}\right.\right) $$, *b* is a possible value (i.e.: a realization) of $$ \widehat{\Delta  B} $$, and *I* (·) is the indicator function.

When *N*, the size of the population targeted by the innovative intervention, is calculated over a time horizon of more than a year, the EVPI has to be discounted, weighted by the term $\sum \limits_{k=0}^{k-1}\left(\frac{1}{{\left(1+\tau \right)}^{k.}}\right)$

Where *k* is the time horizon of the calculation (in years) and *τ* is the discount rate.

C. Determining the optimal sample size in the planned randomised trial-based CEA.

Each additional participant included in a cost-effectiveness study induces a decrease of uncertainty in the cost-effectiveness of the innovative intervention, and consequently a decrease in the expected *EVPI*_*n*_.

Our method defines the optimal sample size as the *n* for which the cost of including two (one in each group) additional participants (2*C*_*p*_) becomes equal or higher than the *EVPI*_*n*_ decrement due to their inclusion (*EVPI*_*n*_ − *EVPI*_*n* + 2_).

Consequently, the optimal sample size is *n* when:

3 $$ \left({EVPI}_{n-2}-{EVPI}_n\ \right)>2{C}_p\  AND\kern0.5em \left({EVPI}_n-{EVPI}_{n+2}\ \right)\le 2{C}_p $$

### Development of an R package

In order to ease the use of our method and to facilitate calculations, we developed an R package (EBASS) (https://CRAN.R-project.org/package=EBASS). This package handles the cases of unequal variances or unbalanced groups. The package tutorial explains how to conduct sample size calculation using our method and how to conduct sensitivity analyses according to a defined range of parameters’ values.

### Obtaining the necessary data

Through his publication, Glick provides guidance on ways to obtain data on parameters required for calculating a sample size based on a test statistic, especially when data require to be generated through specific assumptions when evaluating new interventions [10].

Additional parameters are needed for using our method: the size of the target population (*N*), the annual discount rate (*τ*), the time horizon in years (*k*), and the cost per participant included (*C*_*p*_).

The size of the target population can be estimated through prevalence and incidence data from registries, large cohort studies, medico-administrative databases, or surveillance systems. *N* has to be calculated over the entire time horizon used for the estimation of *EVPI*_*n*_. It is usually easier to gather data on the annual number of individuals susceptible to benefit from the new intervention. If this number is expected to be constant over the time horizon, *N* is the product of this time horizon (in years) and the annual number of individuals. The annual discount rate is defined in each country, generally between 3 to 6%. Finite time horizons are recommended because the value of information depends on future changes in technologies, prices, and evidence. Furthermore, because of discounting, the impact of a time horizon over 15 or 20 years on the estimation of *EVPI*_*n*_ is not significant [16].

Estimating the total cost of a trial is mandatory in the process of setting it up. For estimating *C*_*p*_, we propose to calculate the total cost of the planned trial for a given sample size *n*. The cost per participant (*C*_*p*_) is obtained after subtracting fixed costs from the total cost and dividing the remaining cost by *n*.

## Results

### Application

Data were extracted from a sample size calculation computed for a planned randomized trial-based CEA comparing telemedicine to face-to-face care in elderly patients with complicated chronic wounds in nursing homes. The expected per patient cost of telemedicine was 477€. It took into account the mean number of teleconsultation/patient; the duration of a teleconsultation; the cost of the informatics equipment for telemedicine, and their amortization over 3 years; and the expected number of teleconsultations. The expected per patient cost of face-to-face care was 645€ and took into account the mean number of consultation/patient; the proportion of patients needing a consultation with a dermatologist or a day hospitalization in a geriatric department; the cost of consultations and day hospitalizations, and the cost of medical transportations. The difference in mean costs (*∆C*) was therefore − 168€. The difference in mean effect (*∆E*) was 0.04 QALY and corresponded to the minimal clinically significant difference in utility over a one-year time horizon. Sample size calculations in clinical trials are usually conducted based on the minimal clinically meaningful difference [17], especially when very little information on the endpoint is available. This could be the case when developing a trial-based CEA after having implemented a clinical trial where QALY were not measured. The expected standard deviation of costs (*σ*_*C*_) and effect (*σ*_*E*_) were 2100€ and 0.12, respectively, which reflected a high level of uncertainty surrounding the estimation of costs and effect. The ceiling cost-effectiveness ratio (*λ*) was 20,000€/QALY. The coefficient of correlation (*ρ*) between cost and effect was set at 0.1.

Given the incidence of chronic wounds in nursing homes, the expected size of the target population was 52,000/year over a time horizon of 20 years. The annual discount rate was 0.04 as recommended by the French National Health Authority [18].

Based on these data, it is possible to calculate the mean incremental net monetary benefit (*μ*_*∆B*_) and the common variance of individual net monetary benefits in each group (${\sigma}_B^2$).

$$ {\mu}_{\Delta  B}=0.04\times 20\ 000-\left(-168\right)=968\mathit{\text{\EUR}} $$

$$ {\sigma}_B^2=20\ {000}^2\times {0.12}^2+2\ {100}^2-2\times 20\ 000\times 0.1\times 0.12\times 2\ 100=9\ 162\ 000\kern0.5em {\text{\EUR}}^2 $$

The cost of including an additional participant is fixed and depends on the study design. In this case, *C*_*p*_ was 2257.25 €.

The *EVPI*_*n*_ calculated for a range of different *n* are presented in Table 1. As *n* increases, the variability of *∆B* is reduced, resulting in an increase of the cost-effectiveness probability (the probability of *∆B* being positive when its point estimate is positive) and a decrease of *EVPI*_*n*_. Furthermore, the larger the sample size, the slower the decrease in *EVPI*_*n*_. As long as the *EVPI*_*n*_ decrease is higher than *C*_*p*_, there is a benefit of including more participants. When this decrease is lower or equal to *C*_*p*_, it is no longer worthwhile to include additional participants.

**Table 1:** Cost-effectiveness probability, *EVPI*_*n*_, and expected gain for an additional inclusion according to total sample size *(n)*

Total Sample size (*n*)	Cost-effectiveness probability (%)	*EVPI*_*n*_ (€)	EVPI decreasing for an additional inclusion in each group (€)	Expected gain for an additional inclusion in each group^a^ (€)
100	94,51	10,365,256	480,869	+ 476,355
200	98,81	1,289,276	47,695	+ 43,181
300	99,72	216,451	7248	+ 2734
328	99,81	135,950	4434	−81
400	99,93	41,514	1312	− 3203
500	99,98	8595	262	− 4253
600	99,99	1816	55	− 4460

^a^ The monetary gain for an additional inclusion in each group (i.e. 2 participants in this example) is the difference between the EVPI_n_ decrement and the costs induced by the inclusion and follow-up of two additional participants in the study (2257.25 €/participant in this example)

**Fig. 1:**
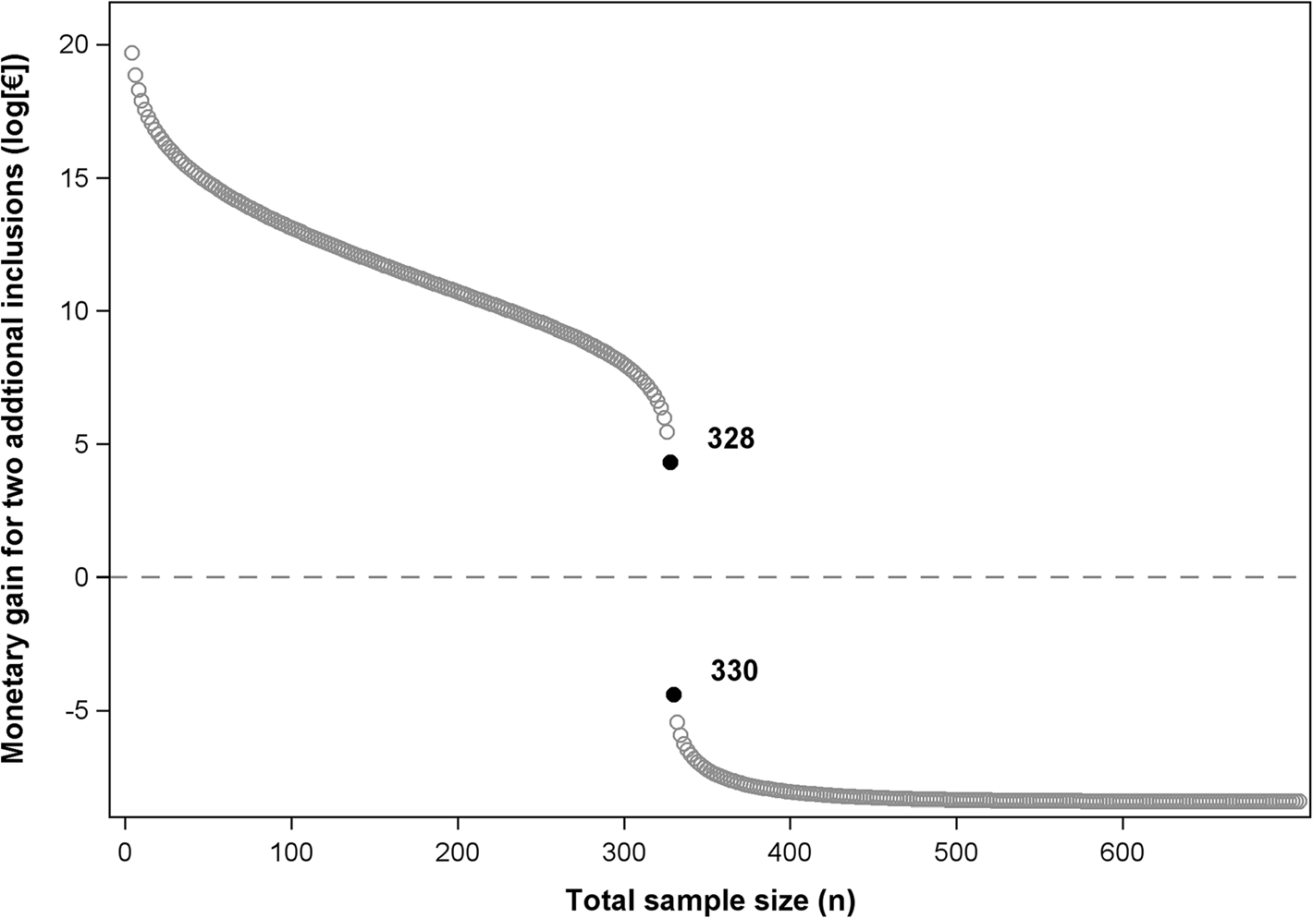
Expected gain from including an additional participant in each group according to the sample size (*n*) (logarithmic scale)

Figure 1 shows the expected gain from including an additional participant in each group, according to total sample size (*n*). This gain, presented on a logarithmic scale, is estimated through the difference between the *EVPI*_*n*_ decrement and the cost of including two additional participants:

$$ \mathrm{Expected}\ \mathrm{gain}=\left( EVP{I}_n- EVP{I}_{n+2}\right)-2{C}_p $$

As shown in Fig. 1, the optimal sample size is reached when any additional inclusion makes this expected gain negative, or when the cost of including two additional participants (2*C*_*p*_) becomes equal or higher than the value of the information gathered through these inclusions (*EVPI*_*n*_ − *EVPI*_*n* + 2_).

The optimal sample size calculated through our method is *n*=328, compared to *n*=306 using a test statistic-based method with a 80% power and a 5% alpha risk. For *n*=328, *EVPI*_*n*_ is 135,950 €, and the cost-effectiveness probability reaches 99.81%.

### Effect of variation of the parameters’ values on *n*

**Fig. 2:**
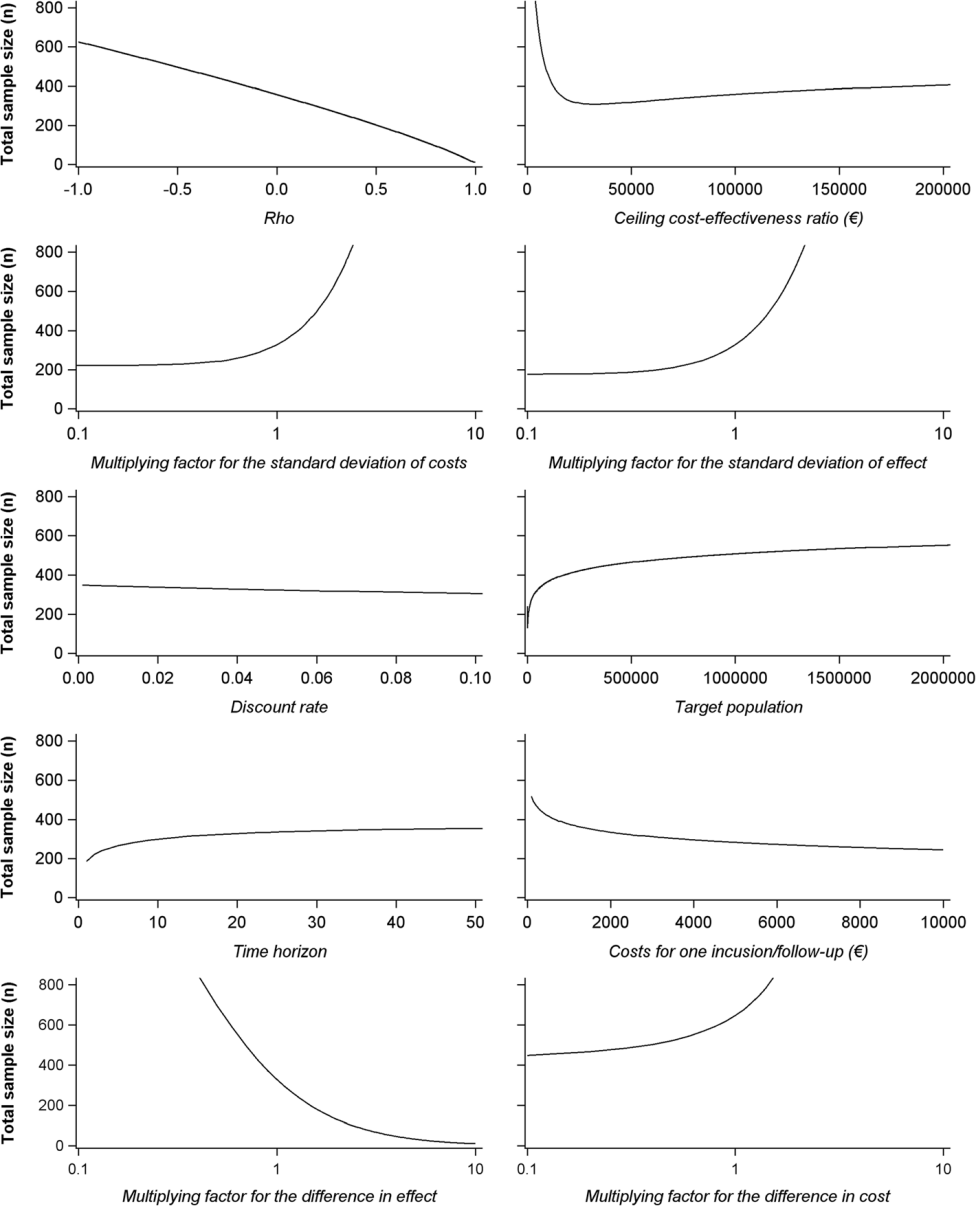
Influence of variation of parameters’ values on total sample size (*n*)

Given the complex relationship between all parameters, variation of the sample size when changing parameters’ values cannot be easily predicted. Figure 2 shows total sample size variations as we vary one by one the parameters of the sample size calculation above: *λ*, *σ*_*C*_, *σ*_*E*_, *ρ*, the size of the target population (*N*), the time horizon (*k*), the discount rate (*τ*), the cost of one inclusion/follow-up, *∆E*, and *∆C*. The variation range for each of these parameters is limited to most plausible values. To facilitate interpretation, variations of *σ*_*C*_, *σ*_*E*_, *∆E*, and *∆C* values range from 0.1 to 10 times the values used in the base-case calculation.

These simulations show which parameters’ inflation increases the sample size (*σ*_*C*_, *σ*_*E*_, *N* and *k*) while other parameters’ inflation decreases it (*C*_*p*_, *τ* and *ρ*). Particular attention should be given to the impact of the value given to *λ* on the sample size calculation results. Firstly and as shown in Fig. 2, when the value of *λ* increases the sample size can either increase or decrease. Secondly, and as shown by Glick using a test statistic-based method, the impact of a variation of *λ* is different according to the value of the other parameters.

To further illustrate our sample size calculation method, we took the example of three cost-effectiveness studies currently conducted with the support of our clinical epidemiology unit, and sponsored by the Bordeaux University Hospital. These three studies have been funded by the French Ministry of Health. The hypotheses used for calculating the sample size of these cost-effectiveness studies are presented in Tables 2 and 3. Lost to follow-up were not considered in these calculations. Sample size calculation results using the method proposed by Glick [10] are reported in Table 2.

**Table 2:** Parameters needed for a sample size calculation method based on a test statistic and resulting sample size per group with the method proposed by Glick

Study	Difference in mean costs (€)	Difference in mean effectiveness	Ceiling cost-effectiveness ratio	Standard deviation of costs (€)	Standard deviation of effectiveness	Coefficient of correlation between the difference in costs and the difference in effectiveness	Sample size per group through the method proposed by Glick ^a^
	(*∆C*)	(*∆E*)	(*λ*)	(*σ*_*C*_)	(*σ*_*E*_)	(*ρ*)	
FEMCAT	312	0,07	16,750 € /complication avoided	100	0,41	0	1000
INTACT	− 725	0,075	20,000 € /QALY	800	0,24	0	75
OXYNAT	17,6	0,00048	100,000 € /complication avoided	1008	0,00078	0	17,350

*QALY* quality-adjusted life year

^a^with a 80% power and a 5% alpha risk

**Table 3:** Additional parameters needed for a sample size calculation method based on the value of information theory and resulting sample size per group with our method

Study	Annual size of the target population	Time horizon (years)	Discount rate	Cost of an additional participant in the study	Sample size per group through our method
	(*N*)	(*k*)	(*τ*)	(*C*_*p*_)	
FEMCAT	670,000	10	0,04	1000	1233
INTACT	20,000	10	0,04	3606	75
OXYNAT	800,000	10	0,04	35	12,951

FEMCAT (Clinicaltrials.gov identifier: NCT01982006) is a multicenter, randomized, pragmatic clinical trial. Its aim is to compare the cost and effect of femtosecond laser-assisted cataract surgery to phacoemulsification cataract surgery. The primary economic endpoint is the cost per complication avoided at 3 months after surgery from the perspective of the French National Health Insurance. The resulting sample size calculated through our method is 1233 eyes per group (Table 3).

INTACT (Clinicaltrials.gov identifier: NCT02599389) is a multicenter triple-arm randomized clinical trial which primary objective is to estimate the incremental cost-effectiveness ratio of two innovative strategies for the treatment of femoropopliteal artery in-stent restenosis: drug-coated balloons (paclitaxel - antimitotic) used alone or in association with the Excimer Laser, both groups being compared to uncoated balloons. The primary endpoint is the cost per Quality adjusted life-years (Qaly) gained at 18 months from the perspective of the French Health System. The sample size calculation was based on the comparison of drug-coated balloons versus uncoated balloons, a comparison that would yield the lowest difference in effect. The result of the sample size calculations using our method is 75 individuals per group (Table 3).

OXYNAT (Clinicaltrials.gov identifier: NCT03078218) is a multicenter controlled before and after study where the cost-effectiveness of pulse oximetry (after), as compared to current medical examination (before), is estimated in the screening of critical congenital heart defects in asymptomatic newborns. The primary endpoint is the cost per complication avoided within a time horizon of 12 months of life from the perspective of the French Health System. The value of the ceiling cost-effectiveness ratio (*λ*) is 100,000 €/avoided complication because death accounts for 20% of complications of critical congenital heart defects. The resulting sample size calculated with our method is 12,951 newborns per group (Table 3).

**Fig. 3:**
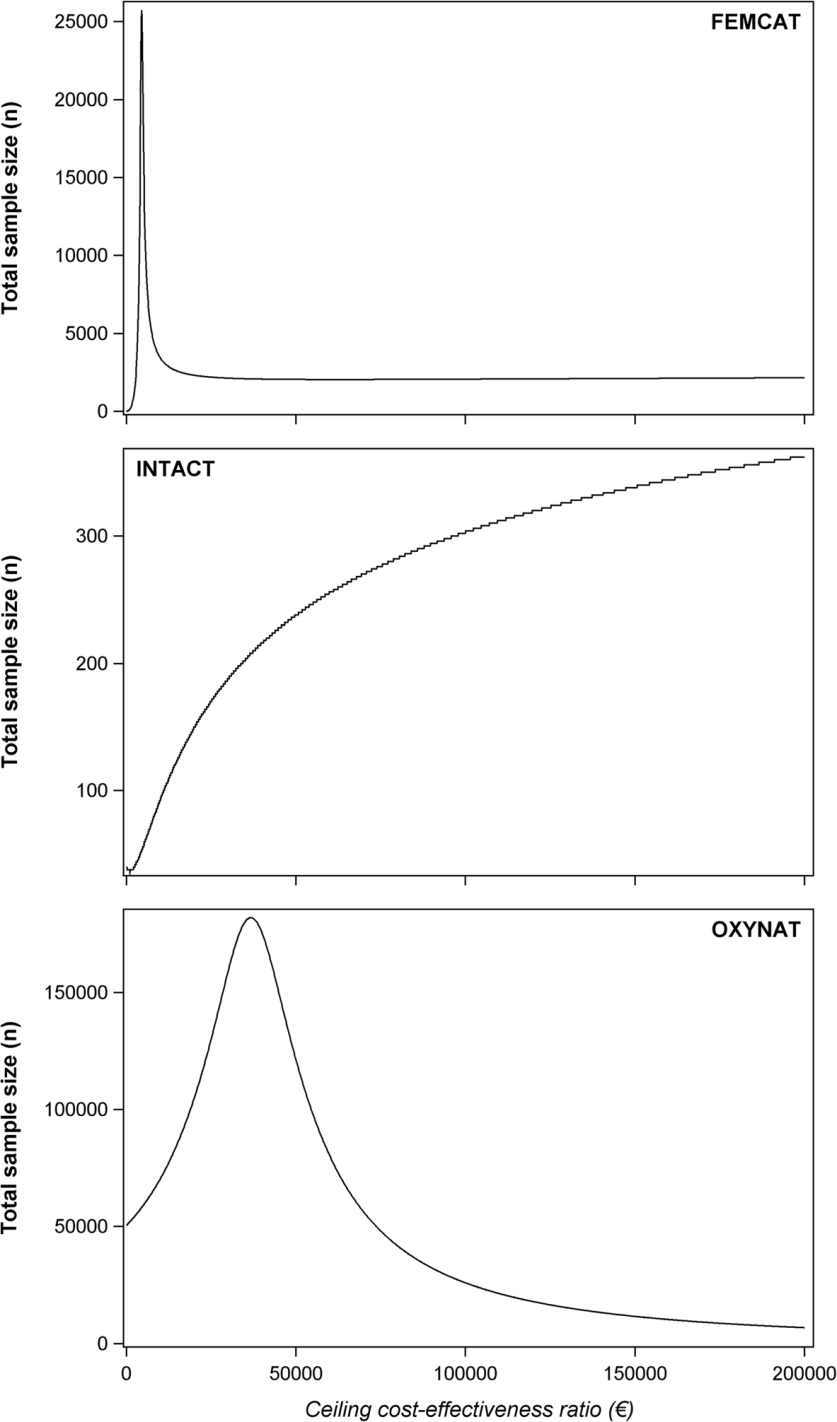
Impact of variation of *λ* on sample size according to three ongoing cost-effectiveness studies described in Tables 2 and 3

Figure 3 shows how widely and differently the sample size can vary for these 3 studies according to the value of the ceiling cost-effectiveness ratio (*λ*). On the x-axis, *λ* values range from 0 to 200,000 €/unit of effect (QALY or avoided complication). The sample size may quickly reach a maximum for the lowest values of *λ* then decrease just as quickly to reach a plateau from 25,000 to 200,000 €/avoided complication as for the FEMCAT study. The sample size may increase with *λ* following a logarithmic shape with stair steps as for the INTACT study. Or it may increase and decrease within a wide range of *λ* values as for the OXYNAT study.

What may explain this variability is firstly that, in the INTACT study, the difference in costs is negative and the difference in effect is positive. Theoretically, the maximum sample size may be reached for a negative value of *λ*. Another explanation is that both the mean incremental net monetary benefit (*μ*_*∆B*_) and the variance of the net monetary benefit (${\sigma}_B^2$) are a function of *λ*. This induces a particularly complex interrelationship between the parameters needed for our calculation method. Additionally, the criterion defining the optimal sample size in our method ((*EVPI*_*n* − 2_ − *EVPI*_*n*_ ) > 2*C*_*p*_ *AND EVPI*_*n*_ − *EVPI*_*n* + 2_ ≤ 2*C*_*p*_) explains why the sample size may increase following stair steps shape according to *λ*.

## Discussion

Our sample size calculation method is perfectly coherent with the decision theory underpinning cost-effectiveness evaluation. It is based on the EVPI which is the first step of a value of information analysis. This criterion is critical as its result helps determining whether further research is necessary or not to reduce the uncertainty in the estimation of the incremental net monetary benefit.

Cost-effectiveness studies typically intervene after (or within) efficacy trials, at the end of the process of evaluating a new intervention. No other trial with the same comparators and targeting the same population should be undertaken after this type of trial. With our sample size calculation method, additional participants should be recruited as long as the expected value of information brought by their inclusion exceeds the cost of their inclusion. Once the sample size calculated through our method has been reached, assuming that the calculation hypotheses are exact, any supplementary inclusion in the same or in a new study aiming at answering the same question would not be worthwhile.

As it is based on the EVPI, our method takes into account the size of the target population. All other parameters remaining unchanged, the lower the size of the target population, the lower the *EVPI*_*n*_, and the lower the resulting sample size. This is of particular added value in the case of rare diseases where patients’ recruitment in clinical trials is a major issue [19].

We only consider a Normal distribution of the incremental net monetary benefit in our sample size calculation method. It is possible that a log-normal or a gamma distribution would be more appropriate and have an impact on the resulting sample size [20]. Further developments of our method and our E-BASS R package could integrate such distributions.

Another possible drawback of our method is the additional number of parameters required for estimating the sample size compared to methods based on test-statistics. They refer to parameters required for estimating the EVPI: the discount rate (*τ*), the size of the target population (*N*), the time horizon (*k*), and the costs of the trial. Hypotheses regarding the value of these parameters have to be made anyway for the recommended value of information analysis of a cost-effectiveness trial. Additionally, estimating the cost of a trial is mandatory in the process of setting it up. Furthermore, there is little uncertainty surrounding these four parameters, and the variations in these parameters’ values change the resulting sample size in limited and predictable ways.

A parameter that may have a great impact on the sample size is the ceiling incremental cost-effectiveness ratio (*λ*). The way variations in the values of *λ* affect the resulting sample size is hardly predictable. Our EBASS R package allows a quick calculation of sample sizes according to a range of plausible values of parameters required for the calculation. This gives the opportunity to choose a conservative sample size when the parameters’ value is uncertain.

We did not consider the question of optimal allocation ratio, which is fixing the sample size and varying the allocation ratio to yield the lowest *EVPI*_*n*_. This is not the aim of our sample size calculation method. Indeed, we consider the allocation ratio as a parameter used in the calculation, as are mean differences in cost and effect and their standard deviations.

An unbalanced allocation ratio is usually decided with regards to ethical considerations, when the effectiveness of the innovation has already been demonstrated (this may be the case for cost-effectiveness trials that usually come at the end of the evaluation process); or in order to improve recruitment, in trials of potentially great public health benefit, where patients may be reluctant to have only 50% chance of receiving the innovation. There are also cases where widespread knowledge about the control intervention exists and more understanding is needed about the innovation. All these situations are taken into account before sample size calculation. We did not see the allocation ratio as an output of our method. However, our methods could be adapted to calculate an optimal allocation as well as an optimal sample size.

Our method follows the same principles than the one proposed by Willan et al. which uses a Bayesian approach [12], while we use a frequentist approach.

When no observed prior distribution of the incremental net monetary benefit is available and that sample size calculations must be computed on hypothesized cost and effect differences, and on hypothesized variance in cost, effect, and their covariance [9, 10], a non-informative prior may be used to run the Bayesian approach. However, in this case, the Bayesian method proposed by Willan et al. and our method may theoretically yield the same sample size. Indeed, in the method proposed by Willan et al., the posterior variance of the incremental net monetary benefit is given by $$ {v}_1=\raisebox{1ex}{$1$}\!\left/ \!\raisebox{-1ex}{$\left(\raisebox{1ex}{$1$}\!\left/ \!\raisebox{-1ex}{${v}_0$}\right.+\raisebox{1ex}{$n/2$}\!\left/ \!\raisebox{-1ex}{$2{\sigma}_B^2$}\right.\right)$}\right. $$[12]. If the prior variance (*v*_0_) tends towards infinity (i.e.: a non-informative prior), $$ \raisebox{1ex}{$1$}\!\left/ \!\raisebox{-1ex}{${v}_0$}\right. $$ would tend towards 0 and *v*_1_ would be equal to $$ \raisebox{1ex}{$4{\sigma}_B^2$}\!\left/ \!\raisebox{-1ex}{$n$}\right. $$, which is the expected variance of the incremental net monetary benefit in a planned study of size *n* in our method.

We think that there is no added value of a Bayesian method in such circumstances.

The results of a decision model or a previous cost-effectiveness study could still be used to run our method. In these situations where a decision could be made based on the results of such studies, the calculation of the size of the target population should take into account the number of individuals to be recruited in the planned cost-effectiveness study and the time the study accrual, follow-up and analysis, as proposed by Eckermann et al [21]

## Conclusions

Value of information is now recognized as a reference for interpreting cost-effectiveness data [5]. Only two sample size calculation methods for cost-effectiveness studies are coherent with this recommendation: ours and the Bayesian framework developed by Claxton [2], and more recently reused by Willan et al [2, 12]. The Bayesian approach seems the most accurate sample size calculation method to use for cost-effectiveness studies when an observed prior distribution of the incremental net monetary benefit is available, such a situation being rare. Our method seems more appropriate when parameters’ values required for the calculation have to be hypothesized.

## Additional file

Additional file 1: Adaptation of our sample size calculation method to the case of unequal variance of costs and effect in each group, and the case of unequal group size. This additional file describes how our sample size calculation method can be used in the case of unequal group size and unequal variances of costs and effectiveness expected in potential samples. (DOCX 16 kb)

## Abbreviations

λ: Threshold monetary value for a unit of effect
*C*_*p*_: Cost per participant included in a study
${\sigma}_C^2$: Variance of costs
${\sigma}_E^2$: Variance of effect
CEA: Cost-effectiveness analysis
ENG: Expected net gain of sampling
EVPI: Expected value of perfect information
EVSI: Expected value of sample information
Δ*B*: Incremental net monetary benefit
*B*: Net monetary benefit
*N*: Size of the target population
*k*: Time horizon in years
*ρ*: Correlation coefficient between cost and effect
*τ*: Annual discount rate
