# Supplementary material for: A new approach for sample size calculation in cost-effectiveness studies based on value of information
Source: BMC Med Res Methodol. 2018 Oct 22;18:113. doi: 10.1186/s12874-018-0571-1 (PMC6198488; doi:10.1186/s12874-018-0571-1)
Supplement: Supplementary file 1 — Adaptation of our sample size calculation method to the case of unequal variance of costs and effect in each group, and the case of unequal group size. This additional file describes how our sample size calculation method can be used in the case of unequal group size and unequal variances of costs and effectiveness expected in potential samples. (DOCX 16 kb) [file 12874_2018_571_MOESM1_ESM.docx]

Additional file

The method proposed in this article is presented for the classical case of equal group size (ratio 1:1) and common variances of costs and effectiveness in each group.

This file describes how our sample size calculation method can be used in the case of unequal group size and unequal variances of costs and effectiveness expected in potential samples.

The theoretical variance of the individual net monetary benefit with the reference intervention (R) and the new intervention (N) are defined as:

$\sigma_{B_{New}}^{2}=\lambda^{2}\sigma_{E_{New}}^{2}+\sigma_{C_{New}}^{2}-2\lambda\rho\sigma_{E_{New}}\sigma_{C_{New}}$ (1.1)

$\sigma_{B_{Ref}}^{2}=\lambda^{2}\sigma_{E_{Ref}}^{2}+\sigma_{C_{Ref}}^{2}-2\lambda\rho\sigma_{E_{Ref}}\sigma_{C_{Ref}}$ (1.2)

Where,

$\sigma_{B_{New}}^{2}$ , $\sigma_{B_{Ref}}^{2}$ are the theoretical variances of the individual net benefit with the new intervention (New) and the reference intervention (Ref)

$\sigma_{E_{New}}$ , $\sigma_{E_{Ref}}$ are the theoretical standard deviations of the effect with the new intervention (New) and the reference intervention (Ref)

$\sigma_{C_{New}}$ , $\sigma_{C_{Ref}}$ are the theoretical standard deviations of costs with the new intervention (New) and the reference intervention (Ref)

$\lambda$ is the ceiling incremental cost-effectiveness ratio

$\rho$ is the correlation between costand effect

Under the hypothesis of independent groups, the variance of the incremental net monetary benefit is defined as:

$\sigma_{\Delta B}^{2}=\sigma_{B_{New}}^{2}+\sigma_{B_{Ref}}^{2}$ (2.1)

Because the group allocation ratio is not necessarily 1:1, $\hat{\Delta B}$, the estimate of $\Delta B$, follows a Normal distribution of mean $\mu_{\Delta B}$ and variance:

$\sigma_{\hat{\Delta B}}^{2}=(\frac{\sigma_{B_{New}}^{2}}{n_{New}}+\frac{\sigma_{B_{Ref}}^{2}}{n_{Ref}})$ (2.2)

where $n_{New}$ and $n_{Ref}$ are the size of each group (new and reference).

Having defined the variance in the sampling distribution of the incremental net monetary benefit in a planned study of unequal group size and unequal variances of costs and effect in each group (see equations above), the sample size calculation method described in our article can be applied. The optimal sample size will then be the $n$ for which $\left[ (EVPI_{n-x}-EVPI_{n})>x\times C_{p} \right] AND [(EVPI_{n}-EVPI_{n+x})\leq x\times C_{p}]$.

The parameter $x$ corresponds to the minimal number of participants to be included with respect to the group ratio (i.e. $x=2$ for a 1:1 ratio; $x=3$ for a 2:1 ratio).
